# Physiology-Guided Management of Acute Right Ventricular Failure and Pulmonary Hypertension in Critically Ill Patients: Hemodynamic Phenotypes, Ventilator Interactions, and Rescue Strategies

**DOI:** 10.3390/medsci14060694

**Published:** 2026-10-21

**Authors:** Akram M. Eraky, Anita J. Chazhikattu, Joseph M. Crum, Jonathan Simmons

**Affiliations:** 1 Division of Critical Care, Department of Anesthesia, University of Iowa Carver College of Medicine, Iowa City, IA 52242, USAjonathan-simmons@uiowa.edu (J.S.); 2 Department of Emergency Medicine, University of Iowa Carver College of Medicine, Iowa City, IA 52242, USA

**Keywords:** right heart failure, TAPSE, RV-PA coupling, pulmonary hypertension, preload-dependent, PFO, ARDS, arterial elastance, systolic right ventricular elastance, sepsis, septic shock, TAPSE/PASP, pulmonary vascular resistance, intrathoracic pressure, inodilators, milrinone, dobutamine, inhaled nitric oxide, epoprostenol, intubation, ARDS, postcapillary PH, precapillary PH

## Abstract

Patients with acute right heart failure or pulmonary hypertension require cautious resuscitation because the right ventricle is highly preload-sensitive and has a narrow therapeutic window; both hypovolemia and volume overload may worsen hemodynamics. Echocardiographic guidance can help optimize fluid administration. The management of right heart failure also includes increasing contractility and reducing systemic afterload with inodilators such as dobutamine and milrinone. In cases of high pulmonary vascular resistance (PVR) contributing to right heart failure, the use of selective pulmonary dilators can decrease the right ventricular afterload, while largely avoiding the systemic hypotension associated with intravenous vasodilators. Hypoxemia, hypercapnia, and acidosis may increase PVR, while sedation, intubation, and positive-pressure ventilation (PPV) may reduce preload, precipitating hemodynamic collapse. When possible, noninvasive respiratory support may be cautiously used to optimize oxygenation and ventilation and avoid intubation. With either form of PPV, airway pressures should be minimized to reduce the risk of hemodynamic compromise. Additionally, preload should be carefully optimized; hypotension-inducing sedatives, such as propofol, should be minimized; vasopressors should be readily available. In ARDS complicated by acute cor pulmonale, a right ventricular-protective ventilation strategy should be considered alongside conventional lung-protective ventilation. This approach emphasizes limiting excessive plateau and driving pressures, avoiding severe hypercapnia and hypoxemia, and carefully titrating positive end-expiratory pressure (PEEP) to prevent excessive increases in PVR and right ventricular afterload. In patients with right-to-left intracardiac shunting, elevated right-sided pressures may worsen shunt flow and cause refractory hypoxemia that responds poorly to increasing FiO_2_ and ventilation pressures. Management should therefore focus on reducing PVR, optimizing right ventricular function, and limiting factors that increase right-sided pressures.

## 1. Introduction

Compared with the left ventricle, which is connected to the high-pressure systemic circulation, the right ventricle is connected to the low-pressure pulmonary circulation, which is designed to accommodate large volumes but not high pressures because of its high compliance. Under normal physiological conditions, the right and left ventricles eject approximately equal stroke volumes. However, because the pulmonary circulation operates at substantially lower pressures and vascular resistance than the systemic circulation, the right ventricle performs approximately one-quarter of the stroke work of the left ventricle. Also, the lower filling pressures of the right ventricle allow coronary blood flow in both diastole and systole, whereas left ventricular coronary flow is mainly in diastole [1,2,3].

Positive-pressure ventilation (PPV) exerts different effects on the two ventricles. Increased intrathoracic pressure decreases preload to both ventricles but may increase right ventricular afterload through increased pulmonary vascular resistance (PVR), while reducing left ventricular transmural afterload. As a result, intubation and mechanical ventilation may precipitate hemodynamic collapse in patients with severe right ventricular contractile dysfunction. In contrast, carefully titrated PPV may improve hemodynamics in patients with left ventricular failure and pulmonary congestion. Table 1 summarizes the physiologic differences between the right and left ventricles [1,2,4,5,6,7].

### 1.1. Right Ventricular-Pulmonary Artery Coupling

If cardiac contractility can overcome PVR and maintain sufficient SV through the pulmonary vascular system, this indicates ventriculo-arterial coupling between the right ventricle and the pulmonary arterial system. This coupling is derived from the systolic right ventricular elastance (E_es_) and arterial elastance (E_a_).

**Figure 1:**
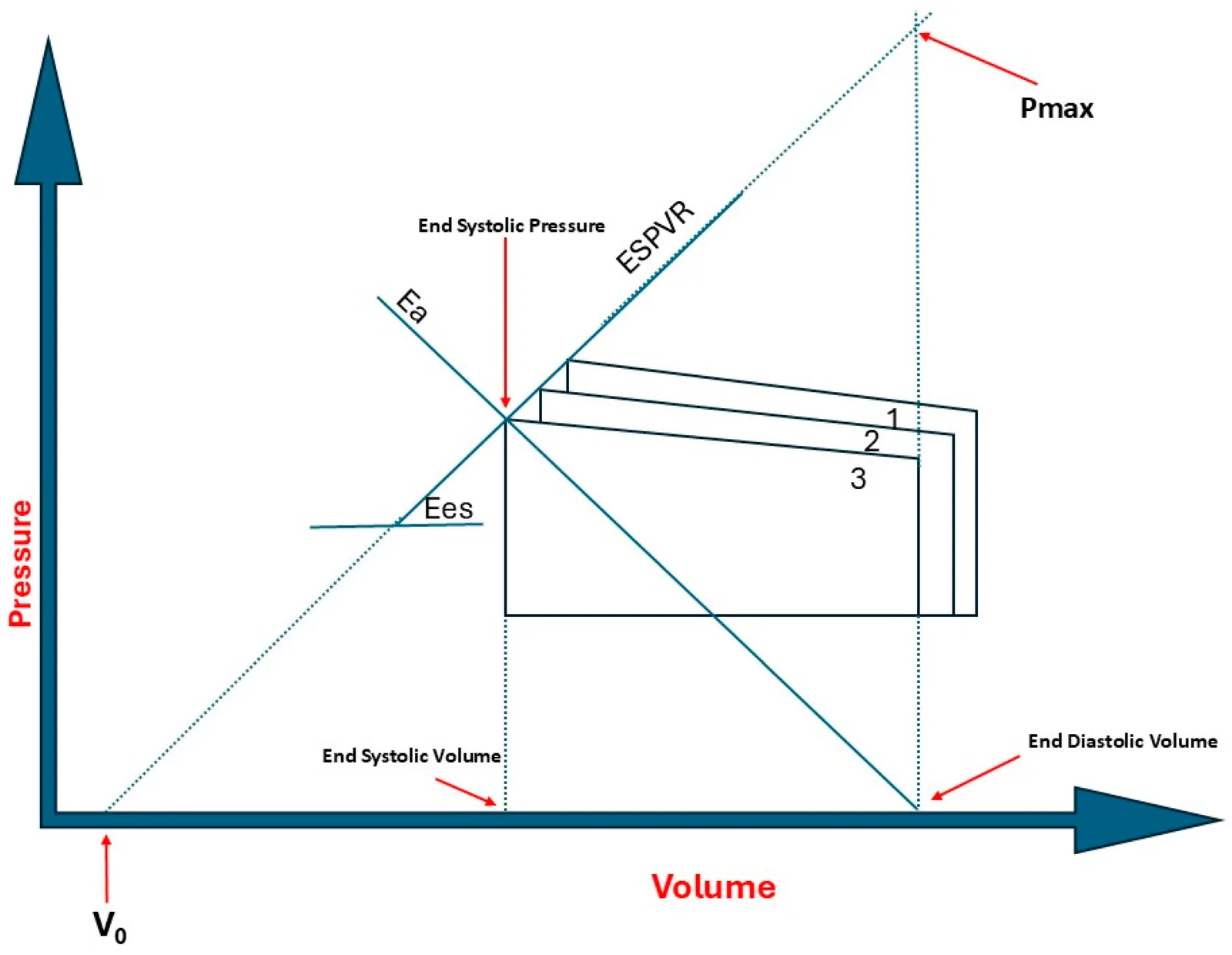
An illustration of the multiple-beat method for obtaining ESPVR with varying preload while maintaining the same contractility and afterload. Abbreviations: E_es_, systolic right ventricular elastance; E_a_, arterial elastance; ESPVR, end-systolic pressure–volume relationship.

The preload-independent ability of the right ventricle to pump is measured by the systolic right ventricular elastance (E_es_), which represents how many mmHg of end-systolic pressure (P_es_) the right ventricle needs to generate for every extra milliliter of volume above the theoretical volume in the ventricle when the pressure is zero (V_0_) [8,9]. E_es_ is calculated invasively by generating multiple pressure-volume loops at different preloads and connecting the different P_es_ points to generate a line. This line is known as the end-systolic pressure–volume relationship (ESPVR). The slope of this line is the E_es_, which represents a preload-independent measure of right ventricular contractility and is expressed in mmHg/mL (Figure 1) [8,9,10,11].

E_es_ can be calculated using two points on the line: point 1 (V_es_, P_es_) and point 2 (V_0_, 0). As a result, E_es_ equals P_es_/(V_es_ − V_0_). After ignoring the theoretical value of V_0_, E_es_ equals P_es_/V_es_ [8]. If a different point is used, including P_max_, which represents a theoretical pressure when the ventricle contracts against the end-diastolic volume (V_ed_) in an isovolumic state without ejecting blood. So, E_es_ can be calculated using two points on the line: point 1 (V_es_, P_es_) and point 2 (V_ed_, P_max_). E_es_ equals (P_max_ − P_es_)/SV [8,11]. E_es_ is influenced by ventricular contractility and stiffness, as it is derived from the change in pressure and volume.

The E_a_ measures the total afterload or the arterial resistance faced by the right ventricle. E_a_ is the slope of the line connecting the P_es_ and the V_ed_; as a result, it will be calculated as the slope between two points: point 1 (V_es_, P_es_) and point 2 (V_ed_, 0). E_a_ equals P_es_/SV. This means E_a_ represents how many mmHg of P_es_ the right ventricle must generate for every milliliter of blood ejected as SV into the pulmonary circulation. E_a_ reflects the pulmonary circulation’s compliance and resistance. It therefore represents the total effective afterload imposed by the pulmonary arterial system on the right ventricle [8,11,12,13,14].

The ratio of E_es_ to Ea represents ventriculo-arterial coupling. When E_es_/E_a_ is 1, this means the right ventricle’s work is maximized. When E_es_/E_a_ is 2, the coupling is very efficient. Given that E_a_ equals P_es_/SV and E_es_ equals P_es_/(V_es_ − V_0_) as discussed earlier, E_es_/E_a_ equals SV/(V_es_ − V_0_). Given that right ventricular SV equals V_ed_ − V_es_ and right ventricular ejection fraction (EF) equals V_ed_ − V_es_/V_ed_, E_es_/E_a_ equals EF/[(1 − EF) − V_0_/EDV]. Given that (V0/EDV) is very small because EDV is always greater than V0, E_es_/E_a_ equals EF/(1 − EF). Given that E_es_ equals (P_max_ − P_es_)/SV, E_es_/E_a_ equals (P_max_/P_es_) − 1 [8].

E_es_/E_a_ of 1.5–2 to 0.8 is considered normal. E_es_/E_a_ < 0.8 is usually associated with the onset of right ventricular dysfunction [15,16]. In a clinical trial, unfavorable right ventricular-pulmonary artery coupling in patients with secondary pulmonary hypertension was defined by a cutoff of E_es_/E_a_ < 0.68 [17].

The challenges of assessing coupling through E_es_/E_a_ include reliance on invasive measures, ignoring the theoretical V_0_, or estimating P_max_. V_0_ has ranged widely and can change with RV dilatation. It therefore cannot always be treated as negligible. Also, EF/(1 − EF) or SV/V_es_ are load-dependent and cannot independently distinguish impaired RV contractility from increased pulmonary arterial afterload [18,19,20]. They can be considered volume-based approximations of RV–PA coupling, while E_es_/E_a_ is load-independent [19].

### 1.2. Non-Invasive Surrogates of the Right Ventricular-Pulmonary Artery Coupling

Getting non-invasive echocardiographic surrogates of E_es_/E_a_ that compare right-ventricular systolic performance with the load imposed by the pulmonary circulation can be more practical [21].

By using the tricuspid annular plane systolic excursion (TAPSE) to represent the right ventricular systolic performance and Pulmonary Artery Systolic Pressure (PASP) to represent the pulmonary systolic pressure load that the right ventricle must generate to eject blood into the pulmonary circulation, TAPSE/PASP is considered a non-invasive echocardiographic surrogate of right ventricular-pulmonary artery coupling. TAPSE/PASP represents the number of millimeters of longitudinal right ventricular shortening achieved for every mmHg of pulmonary arterial systolic pressure that the ventricle must generate. TAPSE/PASP has many limitations, including preload sensitivity, compared with E_es_/E_a_, which are preload-independent. Also, PASP does not independently quantify pulmonary vascular resistance or pulmonary arterial compliance. Patients with similar PASP may have substantially different pulmonary vascular loads. Additionally, TAPSE measures basal tricuspid annular displacement, overlooking radial and circumferential contributions to RV contraction [21,22,23].

A cutoff of TAPSE/PASP of 0.31 mm/mm Hg is considered an indicator of uncoupling, and it represents E_es_/E_a_ < 0.8 [24,25]. In patients with left heart failure, low values of TAPSE/PASP were found to be associated with high mortality and act as an independent predictor of outcomes and mortality, as it indicates the existence of a concurrent right ventricular-pulmonary artery uncoupling [26,27,28,29,30]. In the European Society of Cardiology (ESC) and European Respiratory Society (ERS), TAPSE/PASP was considered a non-invasive measure of right ventricular–pulmonary artery coupling. Also, TAPSE/PASP < 0.55 mm/mmHg was described as a non-invasive finding indicative of pulmonary hypertension [22].

TAPSE can be limited in measuring right ventricular contractility in patients with tricuspid regurgitation and regional wall motion abnormalities [31,32]. In this condition, right ventricular fractional area change (RVFAC) could better represent ventricular contractility. More studies are encouraged to investigate the role of RVFAC/PASP in estimating the coupling [32,33].

## 2. Discussion

This narrative review examines the pathophysiology, hemodynamic consequences, and management of acute right ventricular failure and pulmonary hypertension in critically ill patients, with particular emphasis on cardiopulmonary interactions, fluid management, mechanical ventilation, and hemodynamic support.

The narrative literature review was conducted using PubMed/MEDLINE, Embase, Scopus, and Web of Science. Search terms included “right ventricular failure,” “right ventricular dysfunction,” “pulmonary hypertension,” “acute cor pulmonale,” “RV–pulmonary arterial coupling,” “hemodynamic management,” “fluid resuscitation,” “vasopressors,” “inotropes,” “mechanical ventilation,” “positive pressure ventilation,” “endotracheal intubation,” “pulmonary vasodilators,” and “mechanical circulatory support.” Relevant clinical trials, observational studies, systematic reviews, consensus statements, and international guidelines were considered, with emphasis on contemporary evidence and clinical applicability in critically ill patients.

Evidence from randomized controlled trials, observational studies, systematic reviews, clinical practice guidelines, scientific statements, and expert consensus documents was considered, with emphasis on contemporary evidence and clinically relevant studies.

The review focused on right ventricular physiology and ventricular–pulmonary arterial coupling; diagnostic and hemodynamic assessment; fluid resuscitation; vasopressor and inotropic support; pulmonary vasodilators; and the effects of noninvasive and invasive mechanical ventilation on right ventricular function. Particular attention was given to the physiological consequences of positive-pressure ventilation, peri-intubation hemodynamic instability, and strategies to minimize RV decompensation. Additionally, we highlight the special considerations in the management of critically ill patients with right heart failure, such as patients with septic shock, patent foramen ovale (PFO), and acute respiratory distress syndrome (ARDS).

The available evidence was synthesized narratively, integrating physiological principles with findings from clinical studies and recommendations from professional societies. Areas of uncertainty, conflicting evidence, and limitations in the available literature were highlighted.

### 2.1. Acute Right Heart Failure

Acute right heart failure can be caused by (1) acute increase in right ventricular afterload (pressure overload), (2)acute decrease in contractility, (3) increase in volume in pre-existing right heart dysfunction (volume overload) [2,6,34]. Herein, we discuss the acute increase in right ventricular afterload and the development of deteriorating right heart function in pre-existing pulmonary hypertension or right heart failure.

#### 2.1.1. Acute Pressure Overload-Induced Right Heart Failure

In conditions such as acute pulmonary embolism, ARDS, and severe hypoxia-induced vasoconstriction, patients may develop acute right heart failure due to an acute increase in PVR, which is known as acute cor pulmonale [35]. Pressure overload can also be chronic, due to chronic pulmonary hypertension. The pulmonary vascular system is a low-resistance, high-flow system. The thin-walled right ventricle is designed to pump against low afterload and to compensate well for volume overload, but not very well for pressure overload [5,35,36].

With an acute increase in pressure afterload, PVR and afterload increase significantly, and the right ventricle struggles to compensate against this increased resistance, leading to right ventriculo-arterial uncoupling and right heart dysfunction. Additionally, the right ventricle dilates, shifting the interventricular septum toward the left ventricle and decreasing left ventricular end-diastolic volume and SV [6,37].

#### 2.1.2. Chronic Pressure Overload (Pulmonary Hypertension)-Induced Right Heart Dysfunction

**Figure 2:**
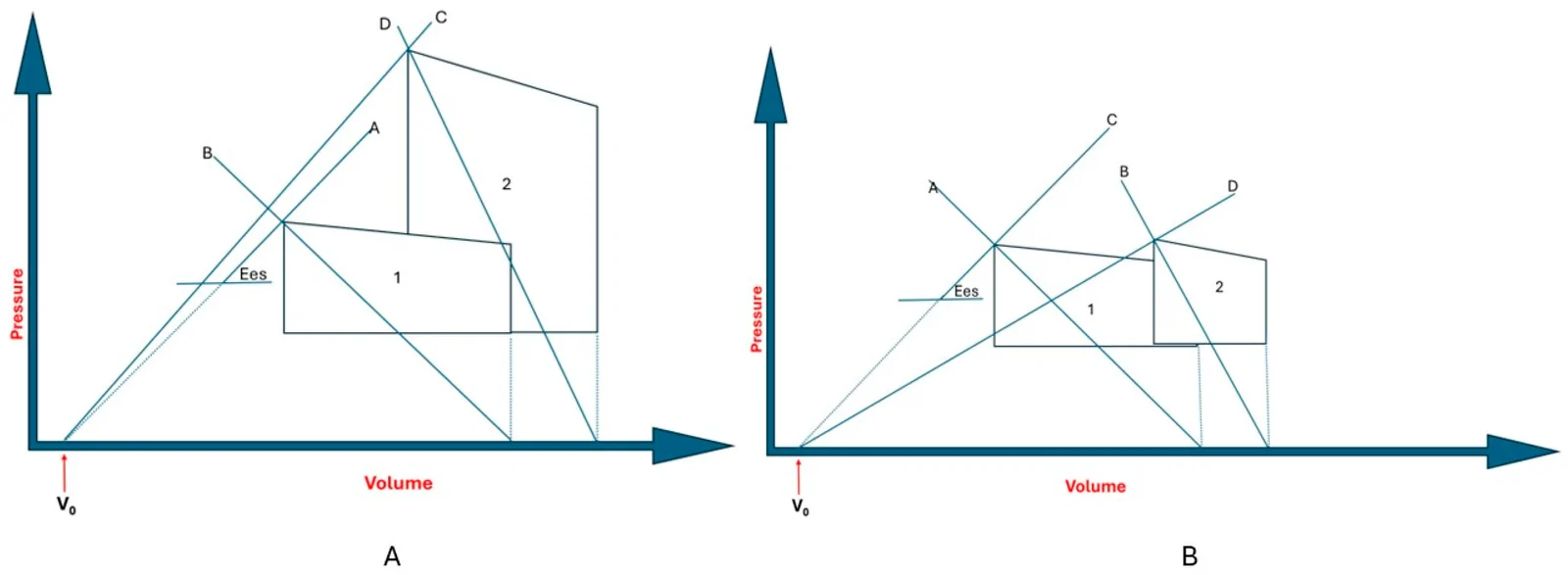
Pulmonary hypertension effect on right ventricular pressure-volume loop. Illustration (**A**) shows the effects of compensated pulmonary hypertension. The pressure-volume loop (1) represents the normal right ventricle, while the pressure-volume loop (2) represents compensated pulmonary hypertension. The pulmonary arterial elastance (E_a_) increases from the slope of line B to the slope of line D. In response to increased E_a_, the RV end-systolic elastance (E_es_) increases from line A to line C as a compensatory reflex. This prevents the E_es_/E_a_ from deteriorating aggressively. Illustration (**B**) shows the effects of decompensated pulmonary hypertension. The pressure-volume loop (1) represents the normal right ventricle, while the pressure-volume loop (2) represents decompensated pulmonary hypertension. The E_a_ increases from the slope of line A to the slope of line B. The E_es_ fails to compensate and decreases due to the severe right ventricular distention from line C to line D.

In chronic compensated pressure overload situations, such as in patients with chronic pulmonary hypertension, decompensation may occur due to volume overload, worsening of pulmonary vascular resistance, or further decreases in contractility [34,38]. In compensated pulmonary hypertension, increased E_a_ is matched by a proportional increase in E_es_, filling pressures, and stroke work, maintaining right ventricle-pulmonary artery coupling and sufficient SV. With decompensation, right ventricular contractile reserve becomes exhausted; E_es_ fails to increase in proportion to E_a_, causing uncoupling, severely increased right ventricular end-systolic and end-diastolic volumes, elevated filling pressures, and reduced SV. On the pressure–volume plane, this might manifest as a rightward shift and narrowing of the previously pressure-loaded loop, as illustrated in Figure 2 [16,22,33,34,36].

Pulmonary hypertension is classified into 5 groups according to the etiology: group 1 is due to pulmonary arterial hypertension, group 2 results from heart disease, group 3 is a consequence of chronic lung disease, group 4 is due to chronic thromboembolic disease, and group 5 is due to unclear multifactorial mechanisms [39].

**Figure 3:**
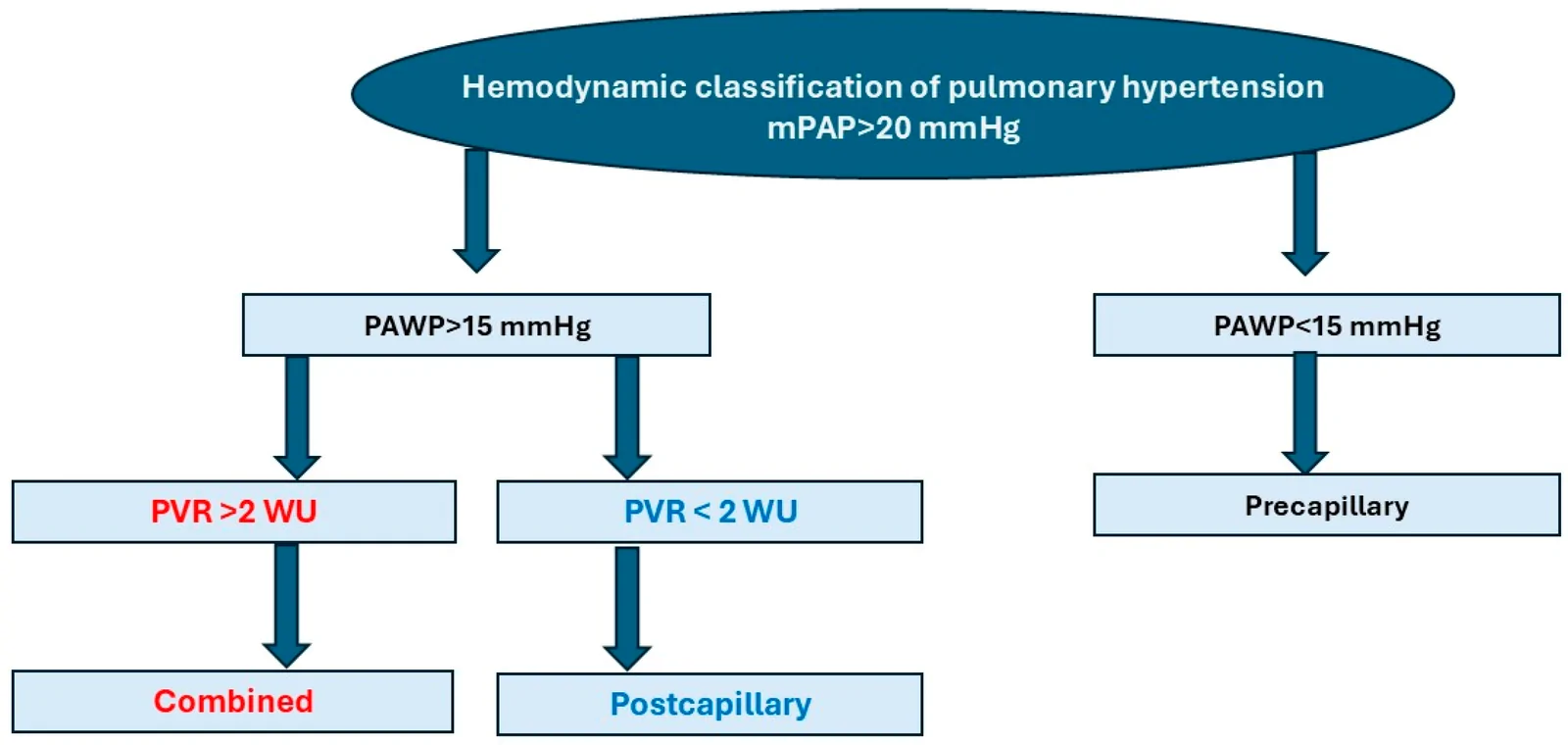
Hemodynamic classification of pulmonary hypertension. Abbreviations: mPAP, mean pulmonary arterial pressure; PVR, pulmonary vascular resistance; PAWP, pulmonary arterial wedge pressure.

From a hemodynamic standpoint, pulmonary hypertension is classified into (1) pre-capillary, in which the pulmonary arterial wedge pressure (PAWP) is ≤15 mm Hg, which is very common in patients with group 1 and group 3; (2) post-capillary, in which the PAWP is >15 mm Hg, which usually represents patients with group 2; and (3) combined pre-and post-capillary, in which PAWP is >15 mm Hg and PVR > 2 WU. To differentiate between pure post-capillary and combined pulmonary hypertension, the PVR should be calculated, defined as the transpulmonary gradient (mPAP-PAWP) divided by cardiac output (CO) (Figure 3) [40,41,42].

The right ventricle’s changed anatomy and dysfunction affect the left ventricle’s functionality. This phenomenon is called ventricular interdependence. Increased pulmonary afterload and pressure load increase wall tension, triggering remodeling. This results in right ventricular hypertrophy and subsequently right ventricular distention due to a continuous increase in pulmonary afterload and end-diastolic volume. This causes the interventricular septum to shift towards the left ventricle, resulting in a decrease in left ventricular end-diastolic volume and, subsequently, a decrease in SV [38,43,44]. Also, the increased oxygen demand by the hypertrophied right ventricle, in addition to the decreased SV by the left ventricle, may lead to right ventricular ischemia [38,44].

### 2.2. Fluid Resuscitation in Critically Ill Patients with Right Heart Failure

**Figure 4:**
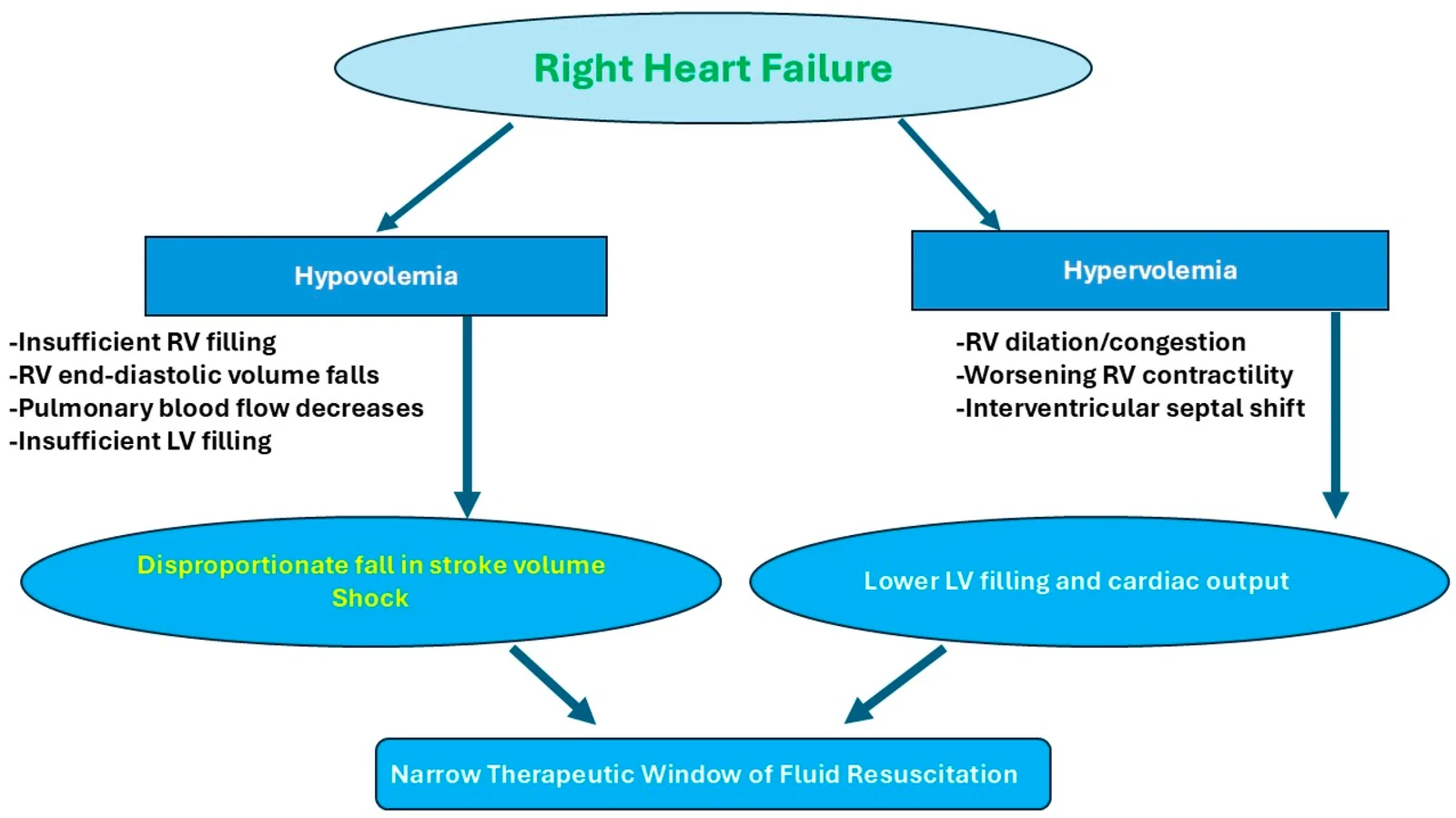
The effect of volume status on the right ventricle. Abbreviations: RV, right ventricle; LV, left ventricle. The RV has relatively limited ability to compensate for an abrupt rise in pulmonary vascular resistance. If stroke volume is already reduced because the RV is ejecting against a high pressure, decreasing preload further may move the patient to the steep portion of the Frank–Starling curve, resulting in a disproportionate fall in stroke volume and instability. In contrast, when the RV is volume overloaded, additional preload may worsen RV dilation, ventricular interdependence, and cardiac output.

Right heart failure is considered a preload-dependent condition, in which the therapeutic window of fluid administration is very narrow [45]. Fluid resuscitation in right ventricular failure should aim to optimize rather than maximize preload; volume expansion may improve cardiac output when right ventricular preload is inadequate, whereas additional fluid in an already dilated, pressure-overloaded right ventricle may worsen ventricular interdependence and systemic venous congestion without increasing SV (Figure 4) [6,36,45].

This preload-dependent concept is well illustrated in a study by Berisha et al., in which the right atrial pressure of 10–14 mmHg was associated with an increase in the right ventricular stroke work index in patients with right ventricular infarction. In contrast, right atrial pressure above 14 mmHg was associated with a decrease in the right ventricular stroke work index [46]. Fluid administration or diuretics in right heart failure should be physiology-guided, based on an understanding of the pathophysiology and echocardiographic findings.

#### 2.2.1. Fluid Resuscitation in Critically Ill Patients with Right Heart Dysfunction Due to Pulmonary Embolism

The European Society of Intensive Care Medicine (ESICM) recommends using surrogate markers of right-heart congestion to guide fluid resuscitation in hemodynamic instability due to pulmonary embolism. The surrogates for right heart dysfunction may include central venous pressure (CVP) > 10 mmHg, echocardiographic findings such as right ventricular size greater than left ventricular size, and Venous Excess Ultrasound (VExUS) Score > grade 1 [47].

The use of diuretics was found to be tolerated in non-hypotensive patients with right heart dysfunction due to pulmonary embolism. In a retrospective study by Ternacle et al., diuresis was associated with a lower shock index, improved systolic blood pressure, and decreased creatinine levels compared with volume expansion in normotensive patients with pulmonary embolism and right ventricular dilation [48].

In an RCT, diuresis improved markers of right ventricular function compared with volume expansion in patients with submassive pulmonary embolism. These markers of improvement in right ventricular function included BNP, troponin, and echocardiographic markers, such as a significant decrease in the right ventricular/left ventricular ratio and an early, lower PASP at four hours [49]. In another RCT, Ferrari et al. found that diuresis was associated with rapid normalization of BNP and was well tolerated in stable patients with intermediate- to high-risk pulmonary embolism [50]. In a similar RCT by Lim et al., diuresis was associated with the absence of oligo-anuria and normalization of the simplified PE Severity Index (sPESI) after 24 h, compared with placebo, in normotensive patients with intermediate- to high-risk pulmonary embolism [51].

#### 2.2.2. Fluid Resuscitation in Critically Ill Patients with Right Heart Dysfunction Due to Myocardial Infarction

In a prospective study by Ferrario et al., fluid resuscitation was associated with increased right atrial and pulmonary capillary pressures in hemodynamically unstable patients with right heart failure due to myocardial infarction. Despite the increase in right-heart pressures, fluid administration did not increase cardiac index, stroke work index, or aortic pressure. In contrast to fluid administration, dobutamine administration was associated with increased cardiac index, stroke volume index (SVI), and right stroke work index. Dobutamine was also associated with a significant decrease in RV end-systolic volume [52]. Other clinical studies showed similar results [46,53,54]. These studies demonstrate that fluid resuscitation might be harmful in right heart failure in the setting of increased filling pressures and right ventricular distention. However, fluid resuscitation could be essential in hypovolemic patients with right heart failure.

#### 2.2.3. Fluid Resuscitation in Septic Patients with Pulmonary Hypertension or Right Heart Failure

In septic patients, mortality increased with pre-capillary pulmonary hypertension severity: approximately 28% in mild, 67% in moderate, and 80% in severe pulmonary hypertension [55].

In a prospective study by Vieillard-Baron et al., larger right ventricular size in mechanically ventilated patients with septic shock was associated with lower fluid responsiveness, as represented by a reduced response to passive leg raising. Of interest, TAPSE was normal in about 60% of patients with echocardiographic evidence of right heart failure, defined as a ratio of the right ventricular end-diastolic area to the left ventricular end-diastolic area greater than or equal to 0.8 [56]. This shows that sepsis-induced cardiomyopathy could cause right heart failure [57,58]. It also draws our attention to the fact that TAPSE can appear normal in right heart decoupling and dysfunction, and that right heart coupling should be measured to assess right heart function [31,56].

In a prospective study by Schneider et al., the non-responders to volume resuscitation showed an increase in right ventricular end-diastolic volume index (RVEDVI) without increases in left ventricular end-diastolic volume index (LVEDVI) or SVI. In contrast, patients who had SVI increase after fluid resuscitation were found to have high RVEDVI and LFEDVI. This could be explained by right heart failure in the non-responders [59].

In contrast to the previous studies, Flynn et al. found that septic patients with pulmonary hypertension received less fluid and more vasopressors. Additionally, those who received less fluid had higher mortality. Of interest, those who did not receive fluids had a delayed start of antibiotics, which could be contributing to the high mortality [60]. This can be explained by the fact that right ventricular failure is a preload-dependent condition. Both mild hypovolemia and mild hypervolemia can cause harm [45].

In the 2012 sepsis guidelines, targeting a CVP of 8–12 mmHg to guide fluid resuscitation was recommended. However, this recommendation was removed in the subsequent sepsis guidelines for 2016, 2021, and 2026 [61,62,63,64]. This can be reasonable when we look at sepsis and septic shock as a medical emergency, and we start treatment early without delay to restore perfusion. However, in some populations, such as right heart failure patients, aggressive initial resuscitation could be harmful and induce cardiogenic shock [45,65]. In patients with right heart failure, having CVP more than 14 was shown to be harmful [46]. Physiology guidance and surrogate markers of volume status, such as echocardiographic evidence of right heart strain or distention, should guide fluid resuscitation in septic patients with right heart failure or pulmonary hypertension (Figure 4) [46,47,60].

### 2.3. Intubation and Ventilation Challenges in Critically Ill Patients with Right Heart Failure

Pre-existing moderate and severe right ventricular dysfunction groups were found to be associated with higher hemodynamic instability and cardiac arrest events after intubation. There was a statistically significant association between in-hospital mortality and Pre-existing severe right ventricular dysfunction [66,67]. Also, acute or decompensating right heart failure is associated with a high risk of cardiac arrest and adverse effects after intubation and initiating mechanical ventilation [68,69].

#### 2.3.1. Intubation in Acute Right Heart Failure Due to Acute Pressure Overload

**Figure 5:**
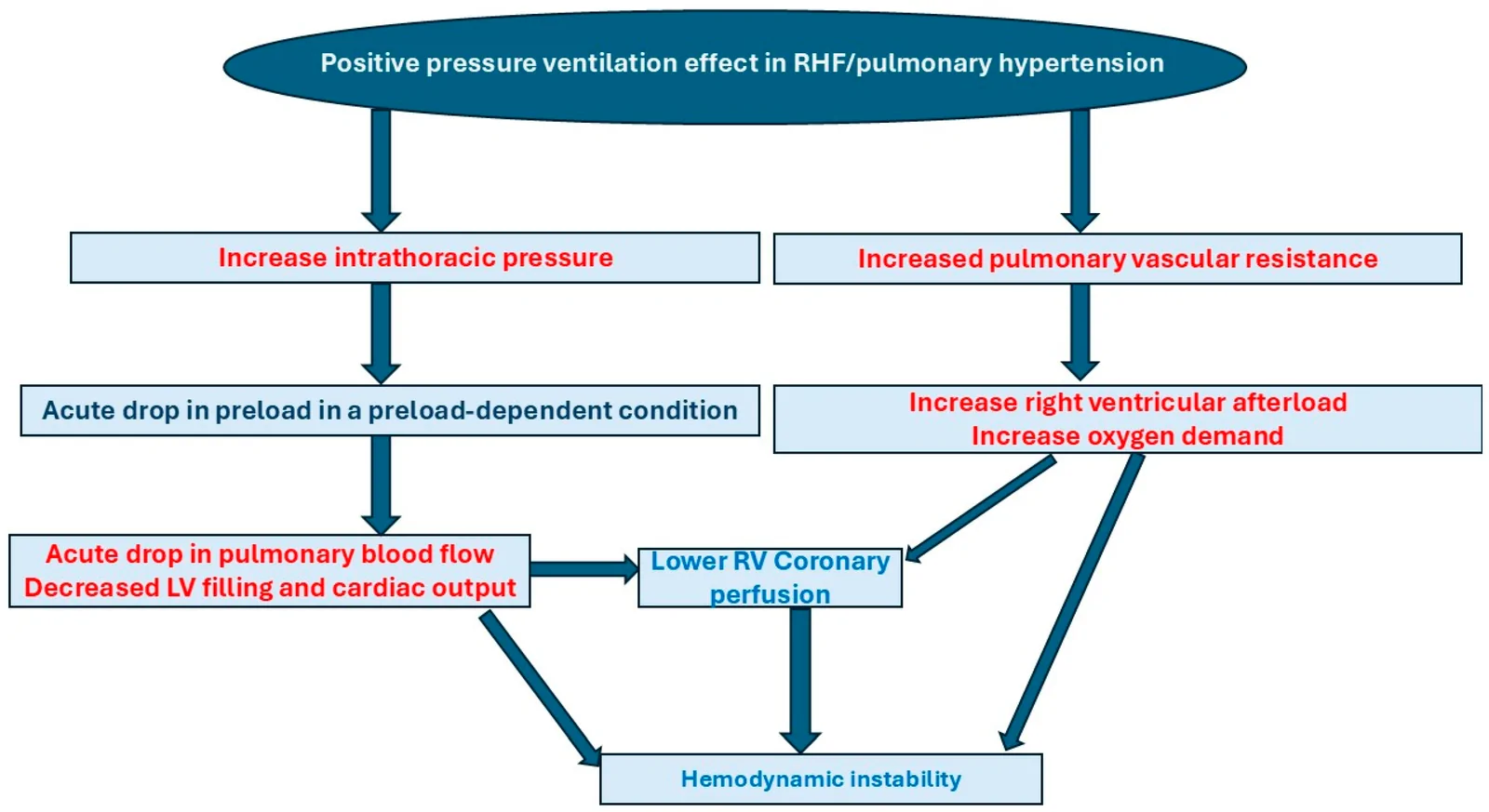
The effect of positive pressure ventilation on the right heart in patients with right heart failure or pulmonary hypertension. Abbreviations: RHF, right heart failure; RV, right ventricle.

In patients with acute right ventricular pressure overload, the right ventricle is not remodeled or hypertrophied and has limited capacity to compensate for the elevated pulmonary afterload. Those patients are sensitive to mild reductions in preload by PPV, sedation-induced hypotension during intubation, and apnea-associated increases in PVR due to hypoxia. This may lead to exacerbation of right heart failure, underfilling of the left ventricle, and subsequently circulatory collapse (Figure 5) [70,71].

Hemodynamic instability is known to be associated with intubation and anesthesia induction in patients with pulmonary embolism requiring embolectomy [70,71,72,73]. In a recent study by Dhar et al., more than half of patients with acute pulmonary embolism were found to experience new or worsening hemodynamic instability after intubation. Right ventricular strain was also associated with increased odds of peri-intubation hemodynamic instability [74]. According to the 2026 multisociety guidelines jointly developed by the American Heart Association (AHA), American College of Cardiology (ACC), American College of Chest Physicians (CHEST), American College of Emergency Physicians (ACEP), and other participating professional societies, deep sedation and mechanical ventilation should be avoided unless clinically indicated in patients with acute pulmonary embolism classified as AHA/ACC Categories C–E, given the risk of hemodynamic collapse [75].

#### 2.3.2. Intubation in Acute Right Heart Failure Due to Decreased Contractility

Acute loss of contractility could result from right ventricular myocardial infarction, myocarditis, or postoperative right ventricular failure. Acute on chronic right heart failure due to decreased contractility could also result from volume overload in a chronic right ventricular myocardial dysfunction due to chronic infarction or ischemic cardiomyopathy [2,76].

Intubation and PPV may precipitate hemodynamic deterioration in right ventricular failure caused by impaired myocardial contractility, even in the absence of severe pulmonary hypertension. Increased intrathoracic pressure reduces systemic venous return and right ventricular preload, while lung inflation may increase PVR and right ventricular afterload. Because the poorly contractile right ventricle has limited reserve, these changes can reduce right ventricular SV, left ventricular filling, systemic arterial pressure, and right ventricular coronary perfusion. Nevertheless, non-invasive or, if necessary, carefully controlled mechanical ventilation may be beneficial when it corrects severe hypoxemia, hypercapnia, acidosis, or excessive work of breathing [2,76].

#### 2.3.3. Intubation in Pre-Existing Pulmonary Hypertension

Chronic pulmonary hypertension may lead to chronic right ventricular dysfunction or failure. Having an acute insult, such as sepsis, pneumonia, pulmonary embolism, or hypovolemia, can lead to exacerbation of the right ventricular failure, resulting in acute right heart failure (Figure 2). Additionally, the management of critically ill patients with pulmonary hypertension can be challenging due to the high risk of hemodynamic instability and developing right ventricular failure following intubation and mechanical ventilation. Mechanical ventilation decreases preload and increases pulmonary vascular resistance, thereby increasing right ventricular afterload [37,77,78,79].

Hong et al. found that having group 1 and group 3 pulmonary hypertension was associated with increased inpatient mortality after intubation, with an odds ratio of 4.03. During the first 24 h after intubation, patients with group 1 and group 3 pulmonary hypertension developed worse outcomes, such as a higher incidence of acute kidney injury and required higher doses of norepinephrine [77]. After intubation, patients with pulmonary hypertension were also found to have a lower PaO_2_/FiO_2_ compared to those without pulmonary hypertension [77].

Gopalratnam et al. found that intubation of critically ill patients with group 1, 3, and 4 pulmonary hypertension is associated with increased in-hospital mortality and 1-year mortality [80]. Also, lower pre-intubation PaO_2_, lower post-intubation PaO_2_, lower post-intubation pH, and higher post-intubation PaCO_2_ were associated with mortality [80]. In a large retrospective study by Rush et al., invasive mechanical ventilation was associated with increased in-hospital mortality up to 39% in patients with pulmonary arterial hypertension [81].

In patients with decompensated pulmonary hypertension complicated by right heart failure, mortality reached 100% with intubation and mechanical ventilation in some studies. In a study by Garcia et al., intubation and mechanical ventilation were associated with 100% in-hospital mortality in patients with decompensated pulmonary hypertension, especially in groups 1 and 4. Of interest, about 54.5% of those patients had a post-intubation cardiac arrest [69]. In another study by Campo et al., the mortality rate was 100% in intubated patients with right heart failure due to decompensated pulmonary hypertension, especially in group 1 [34]. As a consequence of this known high mortality associated with intubation and mechanical ventilation in patients with decompensated pre-capillary pulmonary hypertension, Sztrymf et al. avoided intubating any of their patients in their prospective study [82].

**Figure 6:**
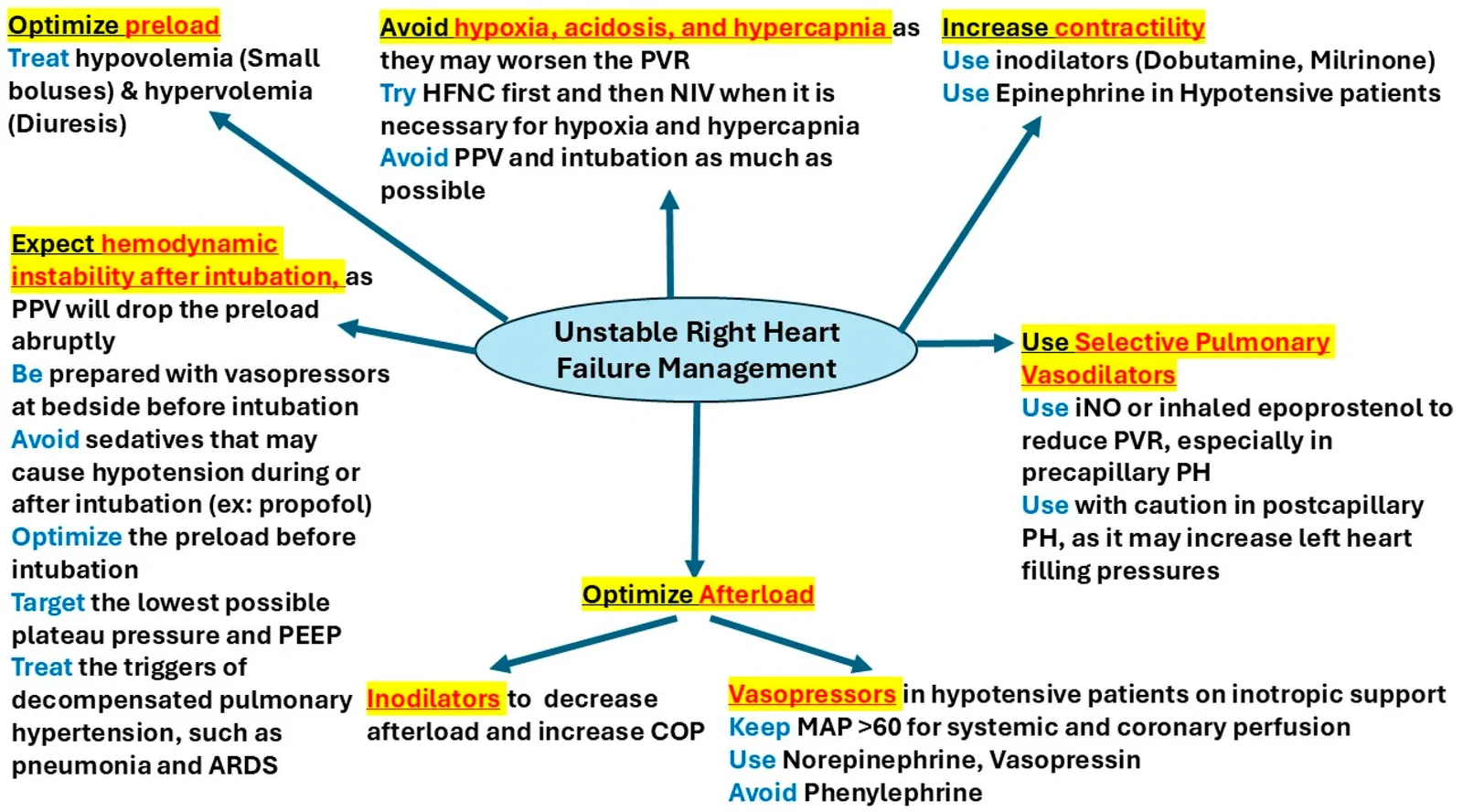
The management of acute unstable right heart failure. Abbreviations: PVR, pulmonary vascular resistance; HFNC, high flow nasal cannula; NIV, non-invasive ventilation; PPV, positive pressure ventilation; iNO, inhaled nitric oxide; PH, pulmonary hypertension; MAP, mean arterial pressure; COP, cardiac output; PEEP, positive end-expiratory pressure; ARDS, acute respiratory distress syndrome.

Management of patients with pulmonary hypertension or right heart failure who may need intubation consists of (1) trying high flow nasal cannula (HFNC) and non-invasive ventilation when it is necessary to treat hypoxemia and hypercapnia and avoiding intubation as long as possible, (2) optimizing preload before intubation as preload is expected to decrease after initiating positive ventilation, (3) expecting hemodynamic instability after intubation and being prepared with vasopressors at bedside before initiating intubation, (4) avoiding sedatives that may cause hypotension during or after intubation, such as propofol, (5) avoiding hypoxia, acidosis, and hypercapnia as it may worsen the elevated pulmonary vascular resistance, (6) targeting the lowest possible plateau pressure and positive end-expiratory pressure (PEEP), and (7) treating the triggers of decompensated pulmonary hypertension, such as pneumonia (Figure 5 and Figure 6) [37,83].

There is a U-shaped relationship between lung volume and pulmonary vascular resistance. Overinflation of the lungs compresses the pulmonary vasculature and increases pulmonary vascular resistance, whereas minimal ventilation may worsen PVR by increasing the atelectatic effect. Non-invasive ventilation may benefit patients who require oxygenation optimization by improving oxygenation, reducing hypoxia- and acidosis-induced vasoconstriction, and decreasing the atelectatic effect [5,37,83,84].

Unlike precapillary pulmonary hypertension, post-capillary pulmonary hypertension is caused mainly by high left ventricular filling pressures and elevated pulmonary venous pressure. In some patients, this leads to functional pulmonary hypertension, defined as pulmonary hypertension not related to vascular remodeling but to elevated left atrial pressure. This type is reversible with decreasing preload with diuresis and decreasing afterload. However, in a subset of post-capillary pulmonary hypertension, patients develop persistent elevation in the PVR even after decreasing the preload and left ventricular filling pressure, which happens when pure post-capillary is transmitted to combined pre- and post-capillary pulmonary hypertension [85,86,87,88].

PPV may provide important hemodynamic benefits in pure group 2 pulmonary hypertension, particularly when acute respiratory failure is driven by elevated left-sided filling pressures and cardiogenic pulmonary edema. By reducing venous return, left ventricular filling pressure, and left ventricular afterload, noninvasive ventilation can reduce pulmonary congestion and improve forward flow. Nevertheless, emergent endotracheal intubation and mechanical ventilation remain hazardous in patients with cardiogenic shock, combined post- and precapillary pulmonary hypertension, or significant right ventricular dysfunction because induction-related hypotension, reduced right ventricular preload, and excessive airway pressures may precipitate circulatory collapse [85]. This reversibility of functional pulmonary hypertension is well demonstrated in patients with aortic stenosis-associated pulmonary hypertension who underwent TAVR. About 45% of the patients experienced resolution of pulmonary hypertension after TAVR [89].

### 2.4. The Dilemma of Ventilation Management in ARDS Patients with Acute Cor Pulmonale or Right Heart Failure

To understand the nuances of ventilator management in ARDS with pulmonary hypertension and right heart failure, we must revisit current guidelines and the tenets of lung-protective ventilation. Current ARDS guidelines recommend lung-protective mechanical ventilation with tidal volumes of approximately 4–8 mL/kg predicted body weight, typically targeting 6 mL/kg, and maintaining plateau pressure below 30 cmH_2_O. Prone positioning is recommended in moderate-to-severe/severe ARDS, particularly with persistent hypoxemia. In moderate-to-severe ARDS, higher PEEP may be considered, but prolonged high-pressure recruitment maneuvers should be avoided. Neuromuscular blockade may be considered in selected patients with early severe ARDS, and venovenous extracorporeal membrane oxygenation (V-V ECMO) should be considered in selected patients with refractory severe ARDS [90,91,92,93].

The European Society of Intensive Care Medicine (ESICM) guidelines recommend against brief high-pressure recruitment maneuvers (defined as airway pressure maintained ≥ 35 cmH2O for less than 1 min) and prolonged high-pressure recruitment maneuvers (defined as airway pressure maintained ≥ 35 cmH2O for at least 1 min) due to the risk of increasing pulmonary vascular resistance, decreasing preload, and developing right heart failure and hemodynamic collapse [91]. Similarly, the updated American Thoracic Society (ATS) and the French guidelines recommend against prolonged recruitment maneuvers [90,93].

The ESICM could not recommend targeting higher or lower PEEP using the high PEEP/FiO_2_ strategy versus the low PEEP/FiO_2_ strategy to reduce mortality in ARDS patients [91]. In contrast, the American Thoracic Society (ATS) guidelines recommend using higher rather than lower PEEP as a conditional recommendation with low-to-moderate certainty [90]. However, in their discussion, they highlighted that higher PEEP may cause lung trauma and hemodynamic compromise [90]. As a result, the net balance of its benefits and harms should be considered. The French guidelines recommend that PEEP be individualized and reserved for improving oxygenation in ARDS patients without worsening hemodynamics or respiratory compliance [93].

In a prospective observational study, acute cor pulmonale developed in 22% of patients with ARDS. Acute cor pulmonale was usually managed with prone positioning and/or nitric oxide. The cor pulmonale group had a significantly higher 28-day mortality rate [94]. In another study, the incidence of acute cor pulmonale was 22.5% in patients with ARDS [95]. As expected, a higher mortality rate was reported in ARDS patients who developed cor pulmonale [94].

When applying the ARDS guidelines in patients with acute cor pulmonale, permissive hypercapnia and acidosis, high PEEP, and high plateau pressures may worsen the PVR and vasoconstriction, resulting in worsening right heart failure and hemodynamic instability. As a result, right ventricular-protective mechanical ventilation emerged as a modification of lung-protective mechanical ventilation for ARDS patients with acute cor pulmonale [96]. This ‘right ventricle-protective’ strategy includes limiting plateau and, in particular, driving pressures; individualized PEEP to balance alveolar recruitment against overdistension; avoiding severe hypoxemia and hypercapnic acidosis; and early prone positioning [91,93,96,97].

In a study by Jardin et al., a plateau pressure of <27 cmH_2_O was associated with a lower mortality rate and incidence of cor pulmonale. Additionally, in patients with cor pulmonale, increasing plateau pressure to higher than 27 cm H_2_O was significantly associated with increased mortality, with an odds ratio of 3.32 [98]. Also, PaCo_2_ ≥ 60 was found to be associated with cor pulmonale with an odds ratio of 3.09, which can be explained by developing hypercapnia-induced pulmonary vasoconstriction [95]. In a prospective study, higher driving pressures were associated with cor pulmonale and higher 28-day mortality. The mean driving pressure in the acute cor pulmonale group was 17 cmH_2_O [94]. In summary, keeping plateau pressure < 27 cmH_2_O, driving pressure < 17 cmH_2_O, and PaCo_2_ < 60 could be right ventricular-protective in patients with ARDS [94,95,98]. More clinical studies are encouraged to investigate other strategies for right ventricular protection in ARDS patients.

Prone ventilation is particularly advantageous in ARDS-induced acute cor pulmonale because it may simultaneously improve oxygenation and CO_2_ clearance, reduce pulmonary vascular resistance, and reverse right ventricular dilatation and septal dyskinesia [99,100]. In refractory cases in which conventional lung-protective ventilation results in excessive driving pressure or severe hypercapnia, extracorporeal gas-exchange strategies may facilitate ultraprotective ventilation and further unload the right ventricle [96]. Table 2 summarizes the differences between (1) lung-protective ventilation in ARDS patients and (2) right ventricular-protective ventilation in ARDS with right heart failure [90,91,92,93,94,95,96,97,98,101].

### 2.5. Patent Foramen Ovale (PFO) in Right Heart Failure: A Cause of Refractory Hypoxia

The foramen ovale does not close in 30% of people and forms a flap-like valve that remains silent and mechanically closed by left atrial pressure. When the right atrial pressure increases sufficiently to overcome the left atrial mechanical closure of the foramen ovale, the valve opens, resulting in right-to-left shunting. This will cause the blood to move from the right atrium to the left heart and bypass the lungs, resulting in persistent refractory hypoxia [102,103,104,105].

The diagnosis of PFO-induced right-to-left shunting in right heart failure patients with refractory hypoxia can be made by performing a bedside echocardiography that may show color flow across the interatrial septum [102]. Also, a bubble-contrast echocardiogram can be beneficial to diagnose PFO in right heart failure without Valsalva provocation [106].

When using positive ventilation in patients with right heart failure and PFO to improve oxygenation, it increases PVR and worsens right-to-left shunting. As a result, the positive ventilation may worsen the patient’s hypoxia. PPV should be avoided in right heart failure patients with right-to-left shunting due to PFO [107,108,109].

Treatment of refractory hypoxia in right heart failure due to PFO should focus on treating the cause of the opening of the right-to-left shunt, which is the right heart failure. Treatment of the reversible causes of right heart failure, such as pulmonary embolism, volume overload, or acute myocardial infarction, should be the priority if it exists. In this situation, PFO is a secondary consequence of the right heart failure [102,110]. Volume overload, unnecessary fluid administration, and PPV should be avoided, as they may worsen right-to-left shunting [111].

In addition to treating potential reversible causes, optimizing volume status and using inotropic support should be considered. Closure of the PFO may worsen right ventricular filling and failure, decrease left ventricular filling, and reduce SV. PFO closure could be considered for patients with refractory right heart failure after failing the other treatment modalities based on multidisciplinary discussions. The use of nitric oxide may be appealing as it may decrease the PVR and preload. However, it may decrease the afterload through systemic vasodilatation; subsequently, it may worsen right-to-left shunting by reducing left ventricular afterload [108,109,110,111,112].

### 2.6. Inotropes and Pressors for Acute Right Heart Failure

Inotropic support may be required to augment the right ventricular contractility. The 2022 European Society of Cardiology (ESC) and the European Respiratory Society (ERS) pulmonary hypertension guidelines specifically identify dobutamine and milrinone as the most commonly used inotropes in patients with advanced right ventricular failure and low cardiac output [87]. Both inodilators, dobutamine and milrinone, can be used to improve right ventricular contractility and decrease pulmonary vascular resistance; however, they carry a risk of systemic hypotension. Also, epinephrine is considered an appropriate inotrope in hypotensive patients. However, it may increase the risk of arrhythmias and tachycardias, as well as increase myocardial oxygen consumption [113,114].

In the case of developing hypotension, norepinephrine is generally the preferred vasopressor because maintaining systemic arterial pressure preserves both systemic perfusion and the right ventricular coronary perfusion gradient, while also providing beta-1-mediated inotropic support. The 2022 ESC/ERS guidelines recommend maintaining a mean arterial pressure (MAP) above 60 mmHg in advanced right ventricular failure. Vasopressors, such as norepinephrine, vasopressin, and terlipressin, can be used when hypotension persists [87,113,114]. Phenylephrine is not preferred as it may cause unopposed pulmonary vasoconstriction and reflex bradycardia [113,115].

### 2.7. Selective Pulmonary Vasodilators for Acute Right Heart Failure

Selective pulmonary vasodilators, such as inhaled nitric oxide (iNO) and inhaled epoprostenol, can be used to reduce pulmonary vascular resistance, especially when elevated PVR substantially increases right ventricular afterload, while largely avoiding the systemic hypotension associated with intravenous vasodilators [113,114,116]. Recent randomized evidence supports this physiologic benefit. In the 2026 PHiNO phase II trial of patients with acute severe right-heart failure due to pulmonary arterial hypertension or chronic thromboembolic pulmonary hypertension, iNO produced significant reductions in the PVR, BNP, and IVC diameter compared to patients who did not receive iNO [117].

Comparative evidence suggests that inhaled epoprostenol may provide similar right ventricular support: in the randomized INSPIRE-FLO trial, inhaled epoprostenol and iNO produced equivalent rates of postoperative right ventricular failure after heart transplantation or left ventricular assist device (LVAD) implantation, with no significant differences in major secondary clinical outcomes [118]. Thus, inhaled pulmonary vasodilators are reasonable short-term therapies for acute right ventricular decompensation associated with elevated PVR, particularly when systemic hypotension limits intravenous pulmonary vasodilators; however, evidence that these therapies improve mortality or other long-term clinical outcomes remains limited [116,119].

Pulmonary vasodilators should be used cautiously in patients with post-capillary pulmonary hypertension or significant left ventricular dysfunction. By reducing PVR and increasing transpulmonary blood flow into a noncompliant or failing LV, these agents may increase left-sided filling pressures, thereby raising left atrial and pulmonary capillary wedge pressure (PCWP). In invasive hemodynamic studies, inhaled nitric oxide increased PCWP in advanced heart failure, while inhaled epoprostenol significantly increased PCWP in patients with heart failure with preserved ejection fraction (HFpEF) [120,121].

Finally, treatment response should not be judged by pulmonary artery pressure alone. Pulmonary artery pressure reflects both pulmonary vascular resistance and the right ventricle’s ability to generate pressure; therefore, a reduction in pulmonary artery pressure does not necessarily indicate hemodynamic improvement and may accompany a decline in right ventricular contractile reserve and forward flow. Treatment response should instead be interpreted by surrogates of right ventricular function, such as CVP, cardiac index, systemic perfusion, and echocardiographic surrogates, such as right ventricle to left ventricle ratio, as well as other measures of right ventricular function and right ventricle–pulmonary artery coupling [87].

### 2.8. Proposed Physiology-Based Clinical Framework for Acute Right Ventricular Failure

Acute right ventricular failure can present in different forms characterized by variable contributions from impaired RV contractility, increased pulmonary vascular afterload, altered preload, and systemic circulatory dysfunction. For practical bedside assessment, we propose four overlapping clinical phenotypes: First, hypotensive right ventricular failure, characterized by reduced systemic arterial pressure and compromised right ventricular coronary perfusion. In this phenotype, vasopressor support, in addition to inotropic support, is important to maintain MAP > 60, avoid systemic hypoperfusion, and improve right ventricular coronary perfusion.

Second, afterload-dominant right ventricular failure is characterized by increased pulmonary vascular resistance and impaired right ventricular–pulmonary arterial coupling. In this type, TAPSE and right ventricular contractility can be normal; the impaired right ventricular–pulmonary arterial coupling is primarily due to increased pulmonary afterload. In this phenotype, a pulmonary-selective vasodilator is highly beneficial. Third, low-output right ventricular failure with preserved systemic blood pressure, in which impaired forward flow may produce tissue hypoperfusion despite the absence of overt hypotension. Fourth, congestion-dominant right ventricular failure, characterized by elevated right-sided filling pressures, right ventricular dilation, and systemic venous congestion. Diuresis in this phenotype has a beneficial role.

These phenotypes are not mutually exclusive and may coexist or evolve during the clinical course. Furthermore, intrinsic RV contractile dysfunction may contribute to several phenotypes independently of pulmonary vascular afterload. This proposed framework is intended to facilitate physiology-guided assessment and individualized management, rather than to establish a validated classification system or to prescribe fixed hemodynamic treatment targets. Figure 6 summarizes the management and special considerations in patients presenting with acute right heart failure.

### 2.9. Mechanical Circulatory Support for Acute Right Heart Failure

In patients with acute right ventricular failure complicated by refractory cardiogenic shock, temporary mechanical circulatory support (MCS) may be considered as a bridge to recovery or transplantation when optimization of preload, reduction in right ventricular afterload, and inotropic support fail to restore adequate systemic perfusion. Table 3 summarizes the guidelines supporting the use of MCS in acute right heart failure with refractory cardiogenic shock [75,87,122,123].

Available devices include percutaneous right ventricular assist devices (RVADs), such as Impella RP and ProtekDuo, surgically implanted RVADs, and V-A ECMO. Device selection should be guided by the underlying pathophysiology, particularly whether right ventricular dysfunction is predominantly due to impaired myocardial contractility or increased pulmonary vascular resistance, as well as by the presence of concomitant left ventricular dysfunction and respiratory failure [35,75,124,125,126,127].

Direct right bypass devices reduce right-sided congestion and restore pulmonary blood flow by transferring blood from the right atrium or right ventricle into the pulmonary artery. However, their effectiveness may be limited in patients with markedly elevated pulmonary vascular resistance, and the resulting increase in left ventricular preload may precipitate pulmonary congestion in patients with concomitant left ventricular dysfunction [35,124,125,126,127].

V-A ECMO may be particularly beneficial in patients with severe pulmonary hypertension, massive pulmonary embolism, or profound circulatory collapse because it bypasses the pulmonary circulation while simultaneously providing systemic perfusion and oxygenation. Nevertheless, VA-ECMO increases LV afterload and may cause LV distension, elevated pulmonary venous pressures, and pulmonary edema, potentially necessitating additional LV unloading. In contrast, in patients with ARDS and right ventricular dysfunction primarily driven by hypoxemia, hypercapnia, and elevated pulmonary vascular resistance, V-V ECMO may indirectly improve RV function by correcting gas exchange abnormalities and facilitating reductions in potentially injurious ventilatory pressures. Table 4 summarizes the different types of MCS that are used in acute right heart failure [35,124,125,126,127].

Prospective clinical studies have demonstrated improvements in hemodynamic parameters and encouraging survival outcomes with temporary right ventricular MCS. However, the evidence remains limited by small sample sizes, predominantly single-arm, nonrandomized designs, and the lack of robust randomized controlled trials demonstrating a mortality benefit. Table 5 demonstrates the current studies supporting the use of MCS in acute right heart failure [128,129,130].

## 3. Conclusions

Patients with acute right heart failure or pulmonary hypertension need special attention during resuscitation. This population is afterload-sensitive, preload-dependent, and has a narrow window of fluid resuscitation; they can be negatively affected by both mild hypervolemia and hypovolemia. Echocardiographic-guided fluid resuscitation is encouraged in this population. Inotropic support should be used in decompensated right heart failure. Vasopressors should be added in hypotensive patients to keep MAP > 60. Selective pulmonary vasodilators should be used if there is increased PVR contributing to the right heart failure.

Intubation, sedation induction, hypoxia, acidosis, hypercapnia, and PPV may cause a sudden drop in preload and acute elevation in pulmonary vascular resistance, resulting in hemodynamic instability. The precautions in the management of right heart failure include: trying HFNC and non-invasive ventilation to correct the hypoxia and hypercapnia only when necessary, avoiding intubation and mechanical ventilation as much as possible, optimizing the preload before intubation, targeting the lowest possible pressures on mechanical ventilation, avoiding sedatives that may cause hypotension during intubation, expecting hemodynamic instability after intubation, and being prepared with vasopressors at bedside before initiating intubation.

## Figures and Tables

**Table 1:** Summarizes the physiologic differences between the right and left ventricles.

Feature	Right Ventricle	Left Ventricle
Normal workload	Pumps into a low-pressure, low-resistance pulmonary circulation	Pumps into a high-pressure systemic circulation
Structure	Thin-walled, compliant, sensitive to acute increases in afterload	Thick-walled and better adapted to pressure loading
Response to increased afterload	Poorly tolerates an acute rise in PVR	Better tolerates moderate acute increases in systemic resistance
Failure from decreased contractility	RV stroke volume becomes highly dependent on adequate venous return and low pulmonary vascular resistance.	LV stroke volume depends on preload, contractility, and systemic afterload; congestion is often prominent.
Effect of induction medications	Vasodilation and loss of sympathetic tone may reduce venous return and systemic pressure, worsening RV output	Can also cause hypotension, but the LV may benefit once excessive systemic afterload is reduced
Effect of positive pressure on preload	Decreases systemic venous return and therefore reduces RV preload	Decreases pulmonary venous return and LV preload after several cardiac cycles
Effect of positive pressure on ventricular afterload	May increase RV afterload by increasing transpulmonary pressure and PVR, especially with lung overdistension	Usually decreases LV transmural afterload because increased pleural pressure reduces the pressure the LV must generate relative to its surroundings.
Effect of PEEP	Too much PEEP can reduce venous return and increase PVR; too little PEEP may permit atelectasis, hypoxemia, and hypoxic pulmonary vasoconstriction.	Moderate PEEP can reduce pulmonary edema, LV preload, and LV transmural afterload.
Effect of hypoxemia, hypercapnia, and acidosis	Particularly harmful because they increase PVR and RV afterload	Can depress contractility and increase sympathetic demand, but they do not directly increase systemic afterload as dramatically as they increase RV afterload.
Potential benefit of ventilation	Correcting hypoxemia, hypercapnia, atelectasis, and excessive respiratory effort may reduce RV stress if airway pressures remain controlled.	Frequently beneficial in LV failure with pulmonary edema by reducing preload, LV afterload, work of breathing, and congestion
Major danger after intubation	Reduced RV preload, increased RV afterload, and decreased systemic pressure can cause abrupt RV output failure.	Excessive preload reduction and induction-related hypotension can decrease cardiac output, particularly in hypovolemia or severe fixed-output states.
Ventricular interdependence	RV dilation shifts the interventricular septum toward the LV, reducing LV filling.	LV output may fall secondarily because of reduced RV output and septal displacement.
Net effect of positive-pressure ventilation	Often unfavorable in severe RV contractile failure unless gas-exchange benefits outweigh the hemodynamic effects	Often favorable in congested LV systolic or diastolic failure, provided hypotension and hypovolemia are absent.

Abbreviations: RV, right ventricle; LV, left ventricle; PVR, pulmonary vascular resistance; PEEP, positive end-expiratory pressure.

**Table 2:** Summarizes the differences between lung-protective ventilation and right ventricular-protective ventilation.

Variable	Lung-Protective Ventilation in ARDS	RV-Protective Ventilation in ARDS + RV Failure/ACP
Primary goal	Prevent ventilator-induced lung injury: volutrauma, barotrauma, atelectrauma	Protect lung + decrease RV afterload + preserve RV output
Tidal volume (VT)	~6 mL/kg PBW; may reduce toward 4 mL/kg if plateau pressure excessive	Usually 4–6 mL/kg PBW, but avoid excessively reducing VT if this causes severe hypercapnia/acidosis
Plateau pressure	≤30 cmH_2_O	Prefer lower plateau pressure when achievable, often conceptually ≤27 cmH_2_O in severe RV dysfunction rather than accepting 30 as automatically “safe”
Driving pressure	Minimize; generally, target as low as possible	Especially important; try to keep <17 cmH_2_O and preferably lower
PEEP	Use enough PEEP to prevent derecruitment and improve oxygenation; moderate/high PEEP often used in moderate–severe ARDS	Individualization based on recruitment AND RV response; avoid both very low and excessive PEEP
PEEP philosophy	“Open/protect the lung”	Find the PEEP producing the lowest pulmonary vascular burden, not simply the best oxygenation
PaCO_2_	Permissive hypercapnia is acceptable if necessary for lung protection	Less permissive hypercapnia; avoid marked rises in PaCO_2_, particularly with RV dilation/ACP
pH	Moderate respiratory acidosis often tolerated	Avoid significant hypercapnic acidemia whenever possible
PaO_2_/oxygenation	Avoid severe hypoxemia while limiting injurious FiO_2_/pressures	Stronger emphasis on preventing hypoxemia
Respiratory rate	Increase RR to maintain minute ventilation during low VT	Increase cautiously; assess auto-PEEP/dynamic hyperinflation
Prone positioning	Recommended early/prolonged in severe ARDS	Strongly favored and can be considered an RV-unloading therapy
Mean airway pressure	Secondary consideration to recruitment and lung protection	Avoid unnecessarily high mean intrathoracic pressure
Rescue strategy	Prone → selected inhaled pulmonary vasodilators → ECMO when indicated	Earlier consideration of RV-unloading rescue, including prone positioning, pulmonary vasodilators and extracorporeal support in refractory cases
Overall philosophy	“Protect the baby lung.”	“Protect the baby lung without overloading the RV.”

Abbreviations: RV, right ventricle; ARDS, acute respiratory distress syndrome; ACP, acute cor pulmonale; PBW, predicted body weight; PEEP, positive end-expiratory pressure; RR, respiratory rate; ECMO, extracorporeal membrane oxygenation; PaO_2_, partial pressure of the arteriolar oxygen; PaCo_2_, partial pressure of the arteriolar carbon dioxide.

**Table 3:** The Guidelines Supporting the Use of MCS in Acute Right Heart Failure with Refractory Cardiogenic Shock.

Society Document	Clinical Setting	Recommendation	Evidence
ESC/ERS 2022 [22]	Advanced PAH with right HF	Selected MCS as a bridge to recovery or transplant (with V-A ECMO being the most common in PAH)	IIa, C
AHA/ACC 2026 [75]	Acute refractory cardiogenic shock due to acute PE	V-A ECMO is reasonable	IIa, B-NR
ISHLT/HFSA 2023 [122]	Acute RV failure	ProtekDuo or Impella RP can be considered	II, B
ISHLT 2023 MCS Update [123]	RV failure in LVAD patients	Early RVAD support should be considered	I, C

Abbreviations: ESC, European Society of Cardiology; ERS, European Respiratory Society; HF, heart failure; PAH, pulmonary arterial hypertension (Group 1 pulmonary hypertension); ECMO, Extracorporeal membrane oxygenation; V-A, veno-arterial; RV, right ventricle; ISHLT, the International Society for Heart and Lung Transplantation; HFSA, Heart Failure Society of America; MCS, mechanical circulatory support; PE, pulmonary embolism; AHA, American Heart Association; ACC, American College of Cardiology; left ventricular assist device, LVAD; Right ventricular assist device, RVAD.

**Table 4:** Different Types of MCS Used in Acute Right Heart Failure.

Device	Mechanism	Best Clinical Application	Major Limitation
Impella RP/RP Flex	Pumps blood from the right-sided circulation into the pulmonary artery	Predominantly isolated RV contractile failure	Does not bypass high PVR or provide oxygenation
ProtekDuo RVAD	Drains right atrial blood and returns it to the pulmonary artery	Isolated RV failure, especially when oxygenation support may be needed, as it is compatible with an oxygenator	Limited effectiveness with severe pulmonary vascular obstruction
Surgical RVAD (e.g., CentriMag)	Extracorporeal RA-to-PA bypass	Severe postcardiotomy or post-LVAD RV failure	Invasive cannulation
VA-ECMO	Drains systemic venous blood and returns oxygenated blood to systemic arteries	RV failure with circulatory collapse, severe PH, massive PE, or biventricular failure	Increased LV afterload and vascular complications
VV-ECMO	Provides extracorporeal gas exchange without directly supporting circulation	ARDS-associated RV dysfunction driven by hypoxemia/hypercapnia	Does not directly support systemic blood flow

Abbreviations: PVR, peripheral vascular resistance; PH, pulmonary hypertension; RA, right atrium; PA, pulmonary hypertension; ECMO, Extracorporeal membrane oxygenation; V-A, veno-arterial; V-V, veno-venous; RV, right ventricle; LV, left ventricle; ARDS, acute respiratory distress syndrome; MCS, mechanical circulatory support; PE, pulmonary embolism; LVAD, left ventricular assist device; RVAD, right ventricular assist device.

**Table 5:** Clinical Studies Evaluating Temporary MCS in Acute Right Ventricular Failure.

Study	Design	Patients (*n*)	Device	Main Findings
Cheung et al., 2014 [128]	Retrospective, two-center cohort	18	Impella RD/RP	Cardiac index increased from 2.1 to 2.6 L/min/m^2^; CVP decreased from 22 to 15 mmHg; 30-day survival was 72%.
Anderson et al., 2015 (RECOVER RIGHT) [127]	Prospective, multicenter, single-arm trial	30	Impella RP	Cardiac index increased from 1.8 to 3.3 L/min/m^2^; CVP decreased from 19.2 to 12.6 mmHg; 30-day survival was 73.3%.
Anderson et al., 2018 [129]	Pooled prospective, nonrandomized studies (RECOVER RIGHT, CAP, PAS)	60	Impella RP	Cardiac index increased from 1.9 to 3.1 L/min/m^2^; CVP decreased from 19 to 13 mmHg; 30-day or discharge survival was 72%.
Anderson et al., 2020 [130]	Nonrandomized comparative analysis (conference abstract)	66	Impella RP	30-day survival was 72.7% in RECOVER RIGHT–like patients versus 15.9% in salvage patients.

Abbreviations: CAP, continued access protocol; CVP, central venous pressure; PAS, post-approval study; MCS, mechanical circulatory support; n, number.

## Data Availability

No new data were created or analyzed in this study. Data sharing is not applicable.
